# Development of a stable chemically cross-linked erythropoietin dimer for use in the quality control of erythropoietin therapeutic products

**DOI:** 10.1007/s00216-019-01768-4

**Published:** 2019-04-10

**Authors:** Paul Matejtschuk, Chinwe Duru, Kiran P. Malik, Adrian F. Bristow, Angele Costanzo, Chris J. Burns

**Affiliations:** 1 National Institute for Biological Standards & Control (NIBSC), Blanche Lane, South Mimms, Potters Bar, Hertfordshire EN6 3QG UK; 2 European Directorate for the Quality of Medicines & HealthCare (EDQM), Council of Europe, 7 Allée Kastner CS 30026, F-67081 Strasbourg, France

**Keywords:** Erythropoietin, Size exclusion chromatography, Dimer, Reference material, Cross-linking, Glutaraldehyde

## Abstract

Erythropoietin (EPO) is a glycoprotein hormone which promotes red cell replenishment and is also a global biotherapeutic medicine widely used to treat anaemia resulting, for example, from chemotherapy. Requirements of the European Pharmacopoeia stipulate that the level of dimer must be quantified in clinical EPO products (with a limit of 2%). Quantification is hampered by the lack of reference preparations containing stable measurable levels of EPO dimer, but the reproducible generation of a stable dimerised EPO preparation is challenging. We describe here the development of a lyophilised, chemically cross-linked EPO preparation, which has good stability and may be used for calibration and system suitability assurance for the size exclusion chromatographic separation of EPO preparations.

**Figure:**
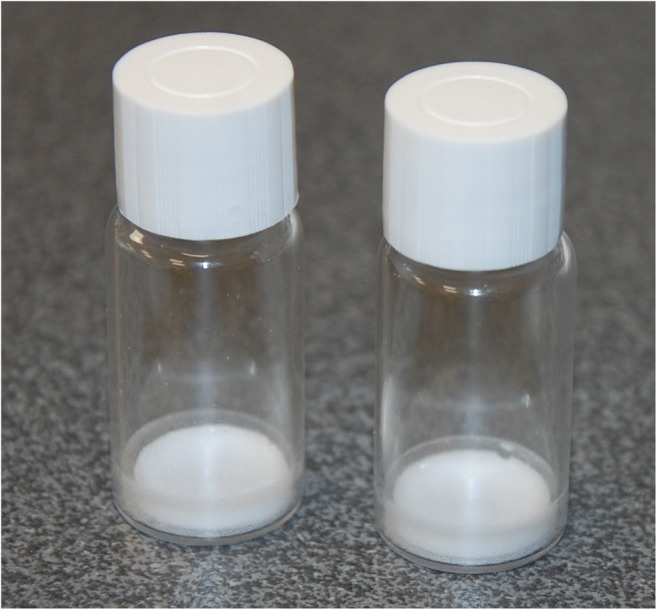
Graphical abstract

Graphical abstract

## Introduction

Erythropoietin (EPO) is a highly glycosylated protein hormone which has its primary effect on red blood cell progenitors and precursors, promoting cell survival and red cell production. Therapeutic EPO products produced by recombinant DNA technology are used to treat anaemia resulting from a variety of medical conditions, and it is also a target of counterfeit medicine and drug abuse. As a result, the characterisation and control of EPO quality and safety attributes is essential to ensure public health.

The importance of high-resolution techniques for the characterisation and control of EPO products has been reviewed by Girard et al. [1]. The European Pharmacopoeia (Ph. Eur.) monograph *Erythropoietin concentrated solution (1316)* prescribes the determination of dimers and higher order oligomers in EPO products and stipulates that dimer content should not exceed 2% by area [2]. Studies have suggested that EPO dimers may form as a result of an initial reduction of internal disulfide bond with subsequent disulfide bond re-oxidation between EPO molecules [3]. Most reference preparations are free from dimers and as such, the analyst performing the test is required to generate their own system suitability sample to ensure that the performance of their method is able to discriminate the dimers and oligomers from monomeric EPO. In-house samples containing dimers and higher order oligomers can be generated by heating monomeric samples [4]. However, the generation of a stable dimeric EPO preparation is not straightforward. Although protocols exist, both the yield and the stability of dimer generated are unpredictable.

In light of these challenges and given the requirement for an EPO preparation with a stable measurable dimer content to aid in the qualification of size exclusion chromatography methods, we initiated an investigation to prepare a stable cross-linked dimeric EPO reference standard. Many cross-linking reagents have been advocated in protein chemistry over the years [5] and have been used widely to probe protein-protein interactions. Similarly, such agents have a long history in the generation of protein complexes for diagnostic use (e.g. immunoassay conjugates). A widely used reagent in this respect, although of less defined chemical specificity than many of the engineered reagents that have followed it, is glutaraldehyde [6]. Here, we report the development of a covalently cross-linked EPO, based upon chemical reactivity of a bridging di-aldehyde reagent—glutaraldehyde—and its suitability for scale-up to prepare a candidate reference material for use as Ph. Eur. Biological Reference Preparation (BRP).

System suitability standards provide a valuable resource for assuring the quality of analytical procedures undertaken by manufacturers and control laboratories. High-performance size exclusion chromatography (HP-SEC) is a very widely used analytical method but confirming the resolving capability of a given column/system is critical if accurate and reproducible data are to be generated.

## Materials and methods

### Erythropoietin

EPO-beta (β) (supplied as a bulk material) was kindly provided by a manufacturer of EPO therapeutic products at 2.5 mg/ml.

### Preparation of highly dimerised EPO

Glutaraldehyde (25% (*w*/*v*) solution, Sigma-Aldrich, Poole, UK) was held at 2–8 °C and fresh dilutions (1/100) made for each dimerisation study. Several molar ratios of glutaraldehyde:EPO were compared, and reaction was allowed to proceed in a controlled temperature environment over a time-course of hours, during which dimerisation was monitored by SEC (see below).

As an example, EPO (1 ml) was thawed, removed aseptically and a 1:3 M ratio of freshly prepared glutaraldehyde stock (0.25 mg/ml, 140 μL) was added and the mixture incubated at 37 °C. After 2 h, a 20-μL aliquot was removed and tested by HP-SEC for dimer formation. A further aliquot was removed after 3- and 5-h incubation. The dimer level rose from 6.5% at 2 h finally reaching 9.27% by 5 h (in terms of peak area). The reaction was then stopped by adding 100 μL of glycine (0.75 mg/ml in water). The sample was then transferred to a 3-ml slide-alyser (Thermo Fisher, Loughborough, UK) and dialysed overnight at 2–8 °C against 200 ml of sodium phosphate buffer pH 7.4. After this, the dialysis buffer was changed to fresh sodium phosphate buffer and dialysis was continued for a further 2 h. The dimerised EPO preparation was then removed (700 μL) and 20 μL tested by HP-SEC with a resultant dimer content of 9.12%.

### High-performance size exclusion chromatography

The principles of the method prescribed in the Ph. Eur. monograph *Erythropoietin concentrated solution* [2] were followed. A TSK 3000SWXL (300 mm × 0.8 mm column, 5-μm pore size) column (Sigma-Aldrich, Poole, UK) was eluted at 0.5 ml/min using a mobile phase of 1.5 mM potassium dihydrogen phosphate, 8.1 mM disodium hydrogen phosphate, 0.4 M sodium chloride, adjusted to pH 7.4 if necessary. A sample injection volume of 100 μl and a run time of a minimum 1 h were used. The detection wavelength was 214 nm. The dimerised EPO (700 μL estimated to contain 1.39 mg EPO, representing 1.26 mg monomer at 9.1% dimer) was diluted to approximately 2% dimer (by area) with monomeric EPO-β in 0.3% (*w*/*v*) arginine, 3% (*w*/*v*) trehalose, 0.01% (*w*/*v*) Tween-20, and 0.45% (*w*/*v*) NaCl, 20-mM sodium phosphate buffer, pH 7.4, to a total EPO concentration of 250 μg/ml.

Filling was performed using a Hamilton 510B dispenser (Esslab Ltd., Westcliffe-on-Sea, UK). The preparation was mixed before starting the filling operations and kept on wet ice throughout. Sixty-one aliquots, 0.4 ml/ampoule, were dispensed into freshly baked (116 °C, approx. 16 h) 3-ml type I glass ampoules which were then part-stoppered for freeze drying using Igloo 13-mm-diameter halobutyl closures.

### Freeze drying

Ampoules were lyophilised in a laboratory dryer (Virtis Genesis 25 EL, Biopharma Process Systems, Winchester, UK) using a ramped freezing rate of 0.4 °C/min to − 40 °C and a primary drying temperature of − 40 °C for 40 h at 30 μbar vacuum. The temperature was then ramped to 25 °C and secondary drying continued for 24 h. Ampoules were stoppered within the dryer after release of the vacuum with dry nitrogen gas, then were flame sealed (OC Ampoule sealer, Adelphi Tubes, Haywards Heath, UK).

### Large-scale preparation of the candidate BRP

This was done by preparing a large bulk of highly dimerised (9.2% by area) preparation of EPO-β, using the method described above, and then diluting it in monomeric EPO-α to give a bulk of 250 μg/ml total EPO.

Filling was performed as 400-μl aliquots into 5-ml screw-capped type I glass vials (18-mm diameter VCD005, Schott, supplied by Adelphi Tubes, UK) using a Bausch & Strobel automated filling line and a CS-150 12 m^2^ Serail production scale freeze dryer (Serail, le Coudray Saint Germer, France). Drying was performed using the same cycle as that described above. The final product was stoppered under dry nitrogen, removed from the dryer and the vials were over-sealed with screw caps on the automated filling line. A collaborative study, involving six laboratories was then organised by the EDQM to determine the fitness for purpose of the candidate material [7].

### Thermal degradation studies

[8] Vials from the initial small fill and from the large batch were stored at defined temperatures over a period of several months and tested for dimer content by HP-SEC, following reconstitution in deionised water, to assess the impact of thermal stress on the lyophilised material.

## Results

Initial studies were undertaken at a EPO:glutaraldehyde molar ratio of 1:3 from a freshly made stock of 0.25% (*w*/*v*) glutaraldehyde in water. Two batches (SS-474 and SS-477) were prepared. Incubation was carried out at 37 °C and aliquots were taken out at 3, 4, and 5 h. Dimerisation was followed in real time by HP-SEC: the dimer content had risen from effectively zero to 1.38% after 3 h and to 8.58% and 9.3%, for SS-474 and SS-477 respectively, after 5 h. The reaction was stopped by addition of glycine. SS-477 was further processed, i.e. dialysed against 100 mM sodium phosphate pH 7.4 and diluted with EPO monomer to give a nominal 2% dimer content (by area) and freeze-dried as described above. A well-formed cake resulted, with a residual moisture content of 1.6% (*w*/*w*) and headspace oxygen of < 0.4%.

Thermally stressed samples were analysed by HP-SEC after 3 months in two different laboratories on different columns (TSK 2000 and TSK 3000). The dimer content was essentially unchanged, even at 45 °C. Both laboratories detected a rise of only 0.1 to 0.25% in the approximately 2% dimer level after around 12 weeks at elevated temperatures (Table 1).

**Table 1:** HP-SEC analysis of a formulated EPO preparation containing chemically cross-linked dimer after storage at elevated temperatures for 12 weeks—mean dimer and monomer contents (in % peak area)

Storage temperature	Analysis on TSK2000SW Lab 1		Analysis on TSK3000SW Lab 2	
	% dimer (*n* = 3)	% monomer (*n* = 3)	% dimer (*n* = 6)	% monomer (*n* = 6)
− 70 °C	2.07	97.93	1.99	97.94
− 20 °C	2.19	97.81	1.92	98.08
20 °C	2.17	97.83	2.05	97.95
37 °C	2.35	97.65	2.14	97.86
45 °C	2.17	97.83	2.09	97.91
EPO CRS	0	100	0.11	99.89

*n* number of runs

No significant difference in the level of dimer was detected in the samples after heat-stress conditions were applied for approximately 12 weeks at temperatures up to 45 °C. The Ph. Eur. EPO for physicochemical tests CRS (not thermally stressed) was tested alongside the samples for comparison and showed no or very low amount of dimer (Table 1).

**Fig. 1:**
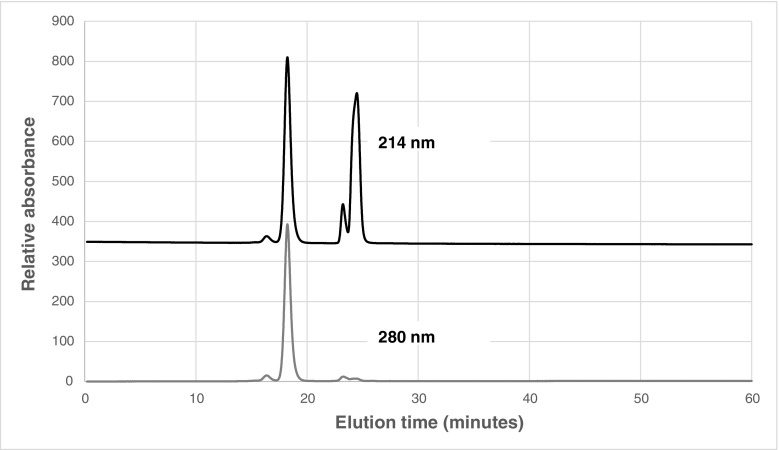
HP-SEC on TSK3000SWXL of formulated EPO preparation (large-scale batch featured) containing cross-linked dimer—monitoring at 214 nm (upper trace) and 280 nm (lower trace). Peaks after 20-min retention are excipient-related. Absorbance values are in relative units and 280nm signal multiplied by 10 to aid visualisation

As the preliminary results were satisfactory, further trials were prepared in screw-capped stoppered vials and subsequently a preparation of a large batch of material containing stable measurable levels of EPO dimer was undertaken in screw-capped 5-ml vials with the aim to establish it as EPO for SEC system suitability CRS. Similar dimerisation, formulation, and lyophilisation methods were used. Additional quantities of glutaraldehyde were required to achieve equivalent quantities of dimerised EPO at the larger scale but a final content of 9% dimer was finally reached. This preparation was then diluted with the monomeric EPO preparation until an approximately 2.4% dimer content was achieved in the desired formulation. However, following analysis of the freeze-dried material, HP-SEC indicated a level of dimer content at 3.63% (by area). The profiles also showed late eluting peaks due to excipient components (Fig. 1). During testing, the quality of the resolution appeared to be influenced by the type and age of the HPLC column used. These findings are further discussed in Matejtschuk et al. [7].

## Discussion

Erythropoietin dimer could be generated by chemical cross-linking using a molar ratio of 1:3 to 1:5 EPO:glutaraldehyde during trial fills. As results of the preliminary tests were satisfactory, a larger batch containing approximately 9% dimer was produced using a molar ratio of 1:3. Following dilution in monomeric EPO to a final dimer content of approximately 2–3% dimer, the bulk material was formulated and freeze-dried in a single large batch (coded 15/120). Following freeze-drying, the ratio of dimer was assessed by HP-SEC and dimer content was assigned at 3.6% (by area) and confirmed qualitatively by migration on reducing SDS-PAGE.

The ability to scale-up the cross-linking process was demonstrated in this study, although estimates of the final level of the dimer content in the preparation proved more variable. Whereas separation achieved before formulation indicated a level of dimer of 2.2%, with one HP-SEC system, it was later shown to be over 3% when measured on a different chromatographic system. This indicates the importance of establishing the performance of the chromatographic column as an indicator of assurance of the derived result.

Following lyophilisation and storage over 2 years at − 20 °C, the dimer content was found to be stable and the consistent resolution of the dimer and monomer forms when analysed by HP-SEC was confirmed. The large-scale batch of material was evaluated by six laboratories across Europe and the results showed in an evaluation of the chromatographic performance of the standard that the promise of the approach was substantiated. The mean dimer content was determined with good consistency across the laboratories (3.4% by area with a CV of 8.2%). The stability post-reconstitution was demonstrated and important lessons drawn about the resolving requirements of the SEC system. The preparation has been adopted as Ph. Eur. EPO for SEC system suitability CRS based on that study [7].

## Conclusion

A preparation containing chemically cross-linked dimerised EPO was produced, dispensed and lyophilised to give a reference material which could be used to qualify the resolving capability of size exclusion HPLC for EPO dimer and monomer.

The level of chemically cross-linked dimer remained constant at the long-term storage temperature. The HP-SEC method for EPO resolved both dimer and higher molecular weight species from the monomer and the approach of generating a system suitability standard for detection of EPO dimer using chemical cross-linking was demonstrated.
